# MicroRNA-128a represses chondrocyte autophagy and exacerbates knee osteoarthritis by disrupting Atg12

**DOI:** 10.1038/s41419-018-0994-y

**Published:** 2018-09-11

**Authors:** Wei-Shiung Lian, Jih-Yang Ko, Re-Weng Wu, Yi-Chih Sun, Yu-Shan Chen, Shin-Long Wu, Lin-Hsiu Weng, Holger Jahr, Feng-Sheng Wang

**Affiliations:** 1 Core Laboratory for Phenomics and Diagnostics, Kaohsiung Chang Gung Memorial Hospital, Kaohsiung, Taiwan; 2 Department of Medical Research, Kaohsiung Chang Gung Memorial Hospital, Kaohsiung, Taiwan; 3 Department of Orthopedic Surgery, Kaohsiung Chang Gung Memorial Hospital, Kaohsiung, Taiwan; 4 Department of Anatomy and Cell Biology, University Hospital RWTH Aachen, Aachen, Germany; 5 Department of Orthopaedics, University Hospital RWTH Aachen, Aachen, Germany; 6 Graduate Institute of Clinical Medical Sciences, Chang Gung University College of Medicine, Kaohsiung, Taiwan

## Abstract

Chondrocyte loss is a prominent feature of osteoarthritis (OA). Autophagy is indispensable in maintaining the metabolic activities of cells exposed to deleterious stress. The contribution of microRNA signaling to chondrocyte autophagy in OA development remains elusive. We uncovered an association between poor autophagy and increased miR-128a expressions in articular chondrocytes of patients with end-stage knee OA and in a rat anterior cruciate ligament transection (ACLT) model for OA development. Cartilage matrix degradation and severe OA histopathology was evident upon forced miR-128a expression within the articular compartment. Intra-articular injections with miR-128a antisense oligonucleotide stabilized chondrocyte autophagy and slowed ACLT-mediated articular tissue destruction, including cartilage erosion, synovitis, osteophyte formation, and subchondral plate damage. In vitro, miR-128 signaling hindered Atg12 expression, LC3-II conversion, and autophagic puncta formation through targeting the 3′-untranslated region of Atg12. It increased apoptotic programs, diminishing cartilage formation capacity of articular chondrocytes. Inactivating histone methyltransferase EZH2 reduced methyl histone H3K27 enrichment in the miR-128a promoter and upregulated miR-128a transcription in inflamed chondrocytes. Taken together, miR-128a-induced Atg12 loss repressed chondrocyte autophagy to aggravate OA progression. EZH2 inactivation caused H3K27 hypomethylation to accelerate miR-128a actions. Interruption of miR-128a signaling attenuated chondrocyte dysfunction and delayed OA development. Our data provide new insights into how miR-128a signaling affects chondrocyte survival and articular cartilage anabolism and highlight the potential of miR-128a targeting therapy to alleviate knee OA.

## Introduction

Osteoarthritis (OA) is the most common cause of joint abnormality, accounts for disability in the elderly and is a huge socioeconomic burden around the world^1^. This disease progressively devastates the entire articular joint compartment, including articular cartilage, subchondral bone, and synovium^2^. Chondrocyte dysfunction is a well-recognized hallmark of the disease and known to accelerate cartilage degradation, synovitis, and subchondral bone remodeling^3–5^. In osteoarthritic chondrocytes, cartilage regulators like TGF-β, IGF, and Wnt signaling components are deregulated, which impairs survival and extracellular matrix (ECM) metabolism^2^. The molecular mechanism underlying the aberrant chondrocyte behavior in OA, however, still remains poorly understood.

Autophagy is an intracellular reaction that preserves protein and organelle functions and shields cells from the detrimental stress augmentation of apoptotic programs^6^. Autophagy participates in the progression of a plethora of disorders^6^. In joint development, autophagy integrates skeletal morphogenesis^7^ and cartilage tissue homeostasis^8^. While the etiological cause of OA remains inconclusive, accumulating evidence now hints towards an association between aberrant autophagy in articular chondrocytes and the development of OA^9,10^. Mice deficient in autophagy regulator Atg5 show chondrocyte apoptosis and histopathological signs of OA in knee joints^11^. Cartilage-specific loss of autophagy repressor mTOR sustains chondrocyte autophagy and prevents aging-induced OA^12^, while Rapamycin activation of autophagy reduces glucocorticoid-mediated chondrocyte apoptosis in OA cartilage^13^.

MicroRNAs (miRs) belong to the small noncoding RNAs that function as critical gene regulators influencing biological or pathological activities through post-transcriptionally targeting mRNA expression^14^. Thousands of mature miRs were identified in human cells and interfere with the translation of a large variety of human proteins modulating tissue metabolism and deterioration^15^. Accumulating evidence links several microRNA pathways also to OA progression. For example, let-7 in serum^16^ and miR-378-5p in synovial fluid^17^ are potent miR signatures in predicting the severity of knee OA. Other miRs, like miR-148a, inhibit expression of key cartilage extracellular matrix (ECM)-degrading enzymes, like MMP13 and ADAMTS5, by osteoarthritic chondrocytes^18^. Chondrogenic TGF-β/Smad signaling^19^ and expression of pro-inflammatory cytokine IL-6^20^ appear to be co-regulated by miR-455-3p and miR-9, respectively. Several studies reveal that abnormal expression of miR-128 is relevant to bone disorders. Forced miR-128 expression reduces migration and epithelial-to-mesenchymal transition capacity of osteosarcoma^21^. Recent studies further show that aging changes the epigenetic status (i.e., DNA methylation signatures) of osteoarthritic chondrocytes^22^. Of note, miR-128 is one of the recently identified top candidates interacting with hypomethylated OA susceptibility genes^22^. Its epigenetic regulation and the crosstalk between miR-128 signaling and autophagic reactions in chondrocytes during OA progression remains elusive.

In this study, we therefore aimed to shed light on the molecular events underlying miR-128a expression, articular chondrocyte autophagy, and OA development. We characterized miR-128a expression in human cartilage, and in an established animal model of surgically induced knee OA. We further used knockdown strategies to evaluate the benefits of miR-128a suppression as an OA modulating therapeutic approach.

## Results

### Elevated miR-128a expression in OA cartilage

**Fig. 1:**
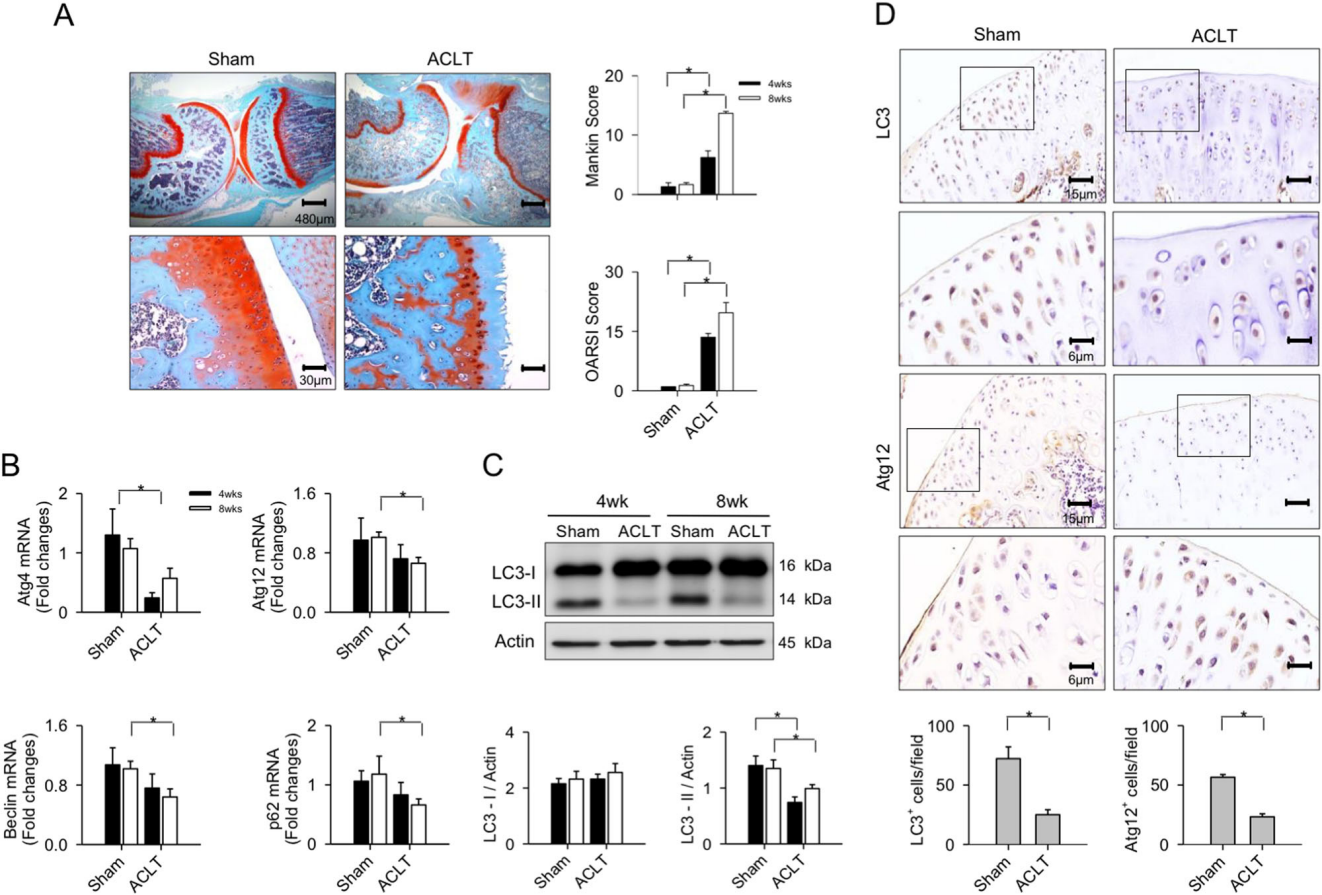
Osteoarthritic changes in knee joints and autophagy marker expression. **a** Histological evidence of articular cartilage degeneration in Safranin-O stained ACLT-affected joints, as compared to sham surgery controls. Mankin score and OARSI score were significantly increased at 8 weeks postoperatively. **b** Decreased expressions of Atg4, Atg12, p62, and Beclin along with **c** reduced LC3-II levels in ACLT-affected articular cartilage. **d** Few chondrocytes in ACLT-injured joints showed Atg12 and LC3 immunostaining. Data per group are expressed as mean ± SEM calculated from eight rats. Asterisks (*) indicate *P* < 0.05 between groups

First, we verified changes in autophagy and microRNA signaling in chondrocytes during OA progression upon anterior cruciate ligament transection (ACLT). Affected rat joints displayed an altered articular cartilage morphology, like microstructural erosion and matrix loss as evident from faint histochemical Safranin-O staining. Consistent with these substantial histopathological changes, ACLT-affected articular compartments further showed a significant increase in Mankin score and OARSI score as compared to the sham group (Fig. 1a). Expression of autophagic markers Atg4, Atg12, Beclin, and p62 (Fig. 1b), and LC3-II, but not LC3-I, levels were significantly reduced in articular cartilage in the ACLT group throughout the study (Fig. 1c). In addition, chondrocytes in the ACLT-injured joints revealed weak LC3 and Atg12 immunostaining (Fig. 1d), which is indicative of defective autophagy in these cells.

**Fig. 2:**
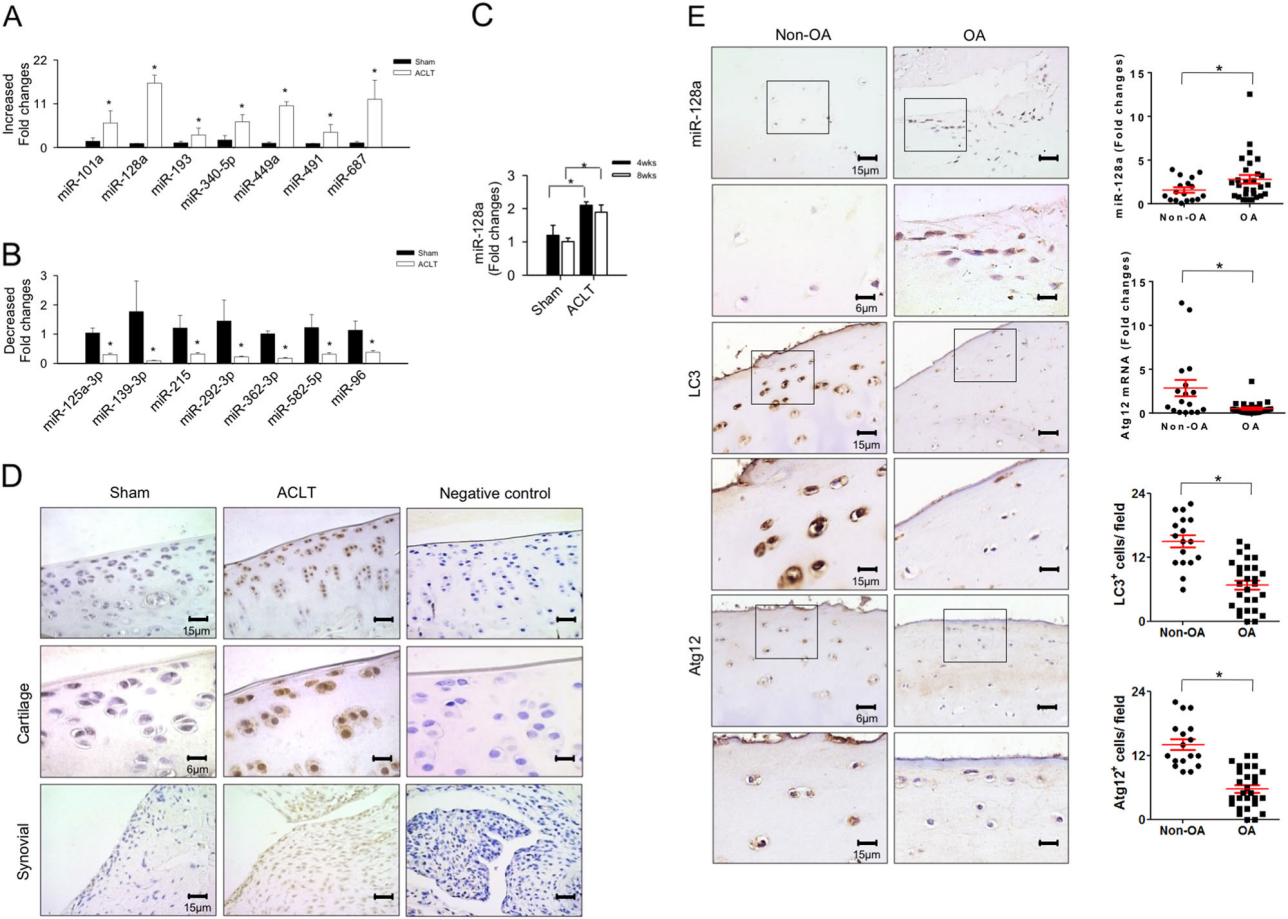
Differential expression of microRNAs and autophagy proteins in osteoarthritic cartilage. **a** Expression of seven microRNAs was significantly upregulated and **b** that of seven other microRNAs significantly downregulated in injured cartilage at 8 weeks after ACLT. **c** Expression of miR-128 was increased throughout the study. **d** In situ hybridization further revealed strong expression of miR-128a in articular chondrocytes and synovial fibroblasts of the ACLT group. Data are expressed as the mean ± SEM calculated from eight rats. Asterisks (*) indicate *P* < 0.05 between groups. **e** Inverse relationship between increased expression of miR-128a and downregulation of Atg12 mRNA in cartilage from patients with end-stage OA. Figures shows in situ hybridization of miR-128a and immunostaining of LC3 and Atg12, respectively (left panels), next to quantification of relative expression of miR-128a and Atg12 mRNA probed by RT-PCR and immuno-intensities in vertical scatter plots (right panels). Data are calculated from 28 patients with end-stage knee OA and 17 patients with femoral neck fracture (non-OA). Asterisks (*) indicate *P* < 0.05 between groups

Comparison of microRNA expression profiles further revealed a significant increase in expression of seven microRNAs (Fig. 2a) in ACLT-injured articular cartilage, while expression of seven other microRNAs (Fig. 2b) was significantly reduced at 8 weeks postoperatively. Expression of miR-128a, which is known to regulate autophagic signaling transduction^23,24^, was most abundantly increased and selected for subsequent experiments. Expression of miR-128a was increased at 4 weeks after ACL transection (Fig. 2c). Histological analyses subsequently confirmed its strong expression by in situ hybridization in chondrocytes and synovial fibroblasts of injured joints (Fig. 2d).

In addition to the increased miR-128a expression in surgically induced OA in rats, specimens harvested from patients with end-stage knee OA also exhibited a significant increase in miR-128a expression along with a decline in Atg12 expression, as compared to the non-OA group. While osteoarthritic chondrocytes showed strong miR-128a transcripts, only few cells stained positive for Atg12 and LC3 immunostaining (Fig. 2e and Fig. S1).

### miR-128a induced spontaneous injury of articular cartilage

**Fig. 3:**
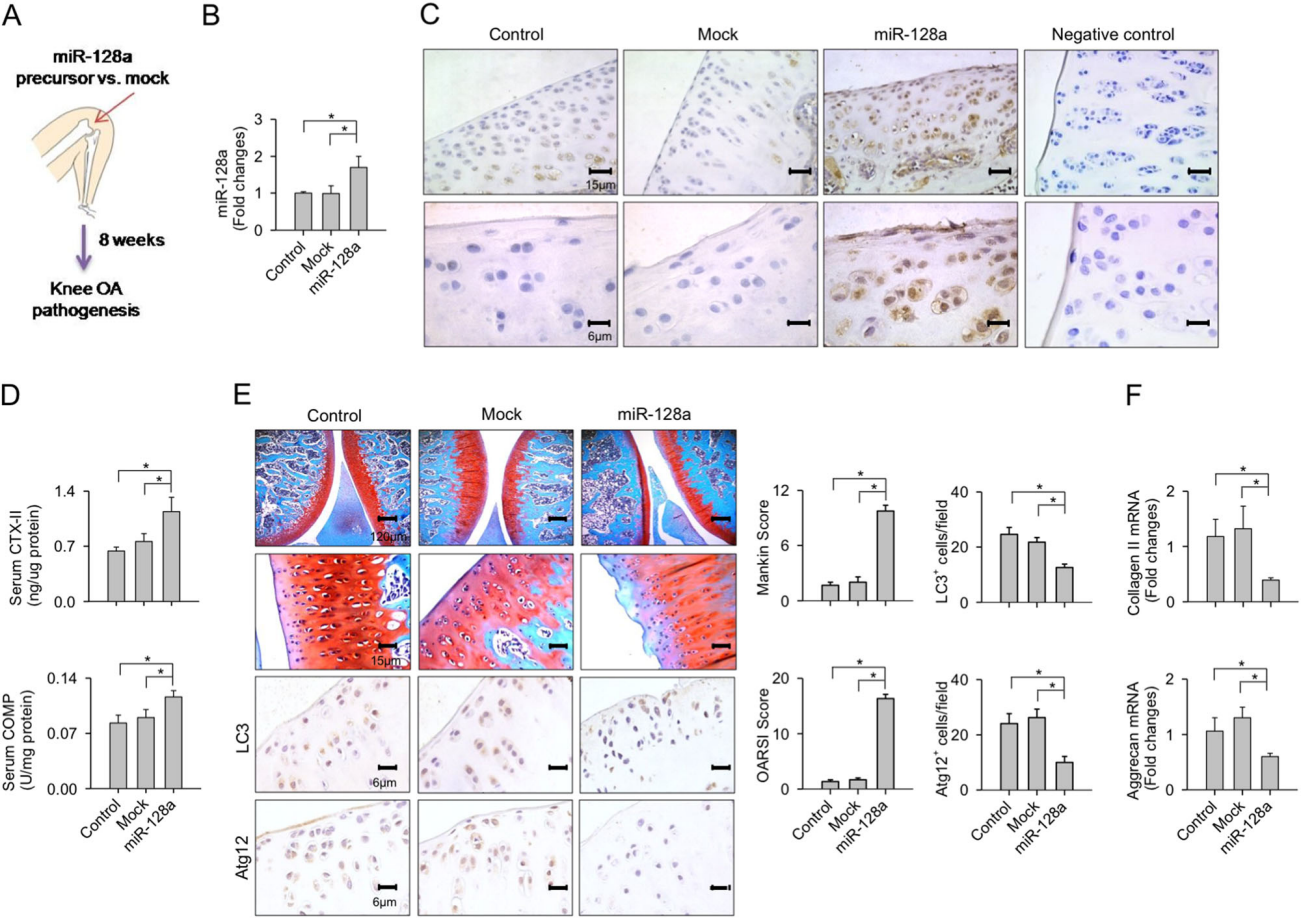
Effects of miR-128a overexpression on articular cartilage integrity. **a** Scheme for intra-articular injection of lentivirus-shuttled miR-128a precursor, **b** showing increased expression of miR-128a, 8 weeks after injection. **c** In situ hybridization upon miR-128a precursor treatment, confirming highly abundant miR-128a transcripts in articular chondrocytes. **d** Increased levels of serum CTX-II and COMP in miR-128a precursor-treated rats. Articular compartments revealing **e** severe cartilage destruction along with weak Atg12 and LC3 immunostaining, and **f** reduced expression of collagen II and aggrecan. Data are expressed as the means ± SEM calculated from nine rats. Asterisks (*) indicate *P* < 0.05 between groups

Intra-articular injection into rat knee joints was used to locally overexpress miR-128a precursors from lentiviral vectors in articular cartilage (Fig. 3a). Expression levels increased by 78% (Fig. 3b) and prominent local miR-128 expression was confirmed by in situ hybridization at 8 weeks after injection (Fig. 3c). Of note, levels of serum markers of cartilage degradation, CTX-II and COMP, were significantly increased in rats at this timepoint as compared to the control group (Fig. 3d). The miR-128a-treated knee joints displayed substantial cartilage erosion along with weak Atg12 and LC3 immunostaining in chondrocytes (Fig. 3e). Expressions of ECM markers collagen II and aggrecan were also significantly reduced (Fig. 3f), while mock treatment did not evidently alter miR-128a expression or cartilage ECM integrity as compared to the normal control group.

### miR-128a interruption alleviated ACLT-mediated cartilage damage

**Fig. 4:**
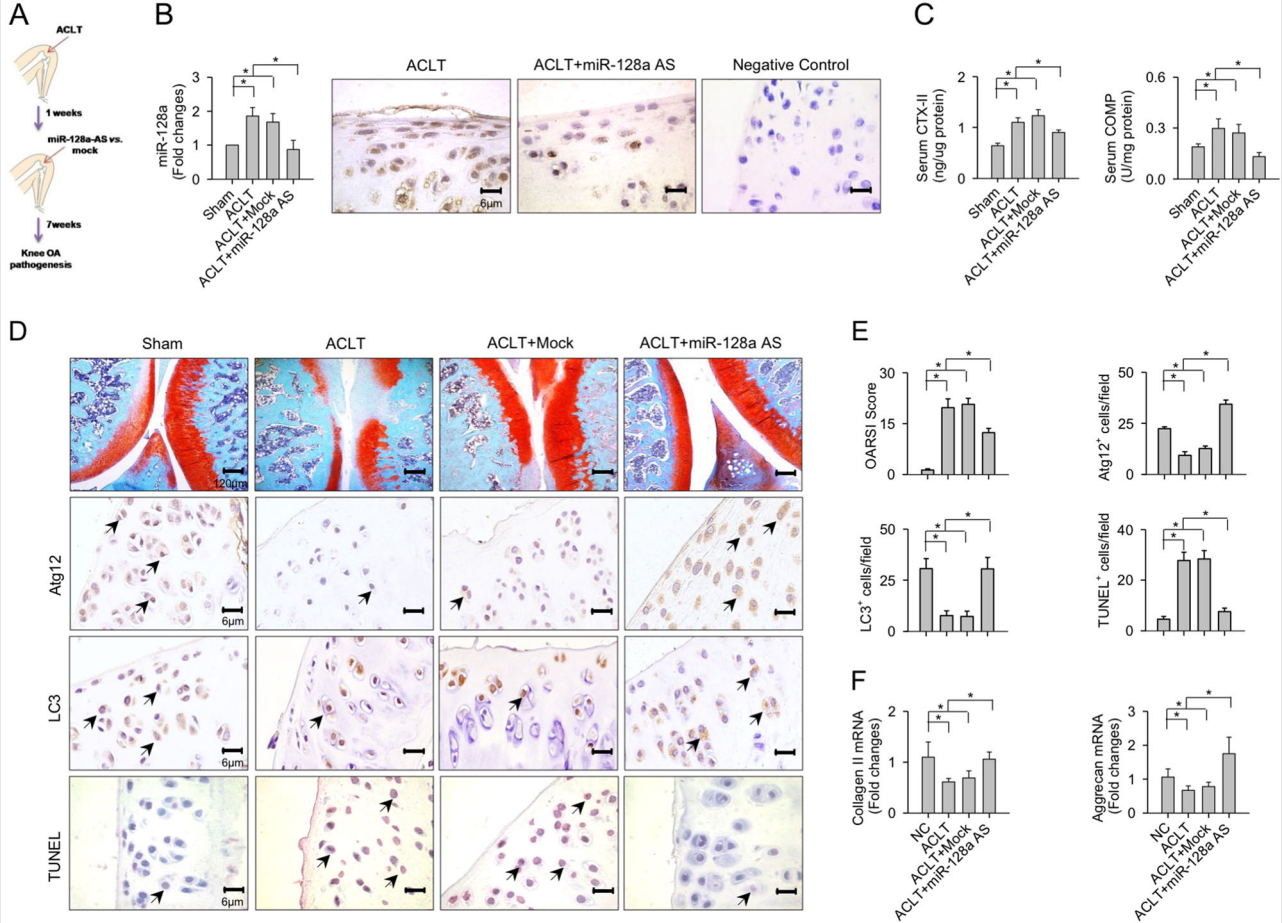
Effects of miR-128a antisense oligonucleotide (miR-128a-AS) treatment on articular cartilage integrity. **a** Scheme of intra-articular injections of miR-128a-AS into ACLT-injured knees. **b** Weak expression of miR-128a transcripts in chondrocytes after antisense treatment and **c** reduced rat serum levels of CTX-II and COMP in ACLT-injured knees. **d** Prominent Atg12 and LC3 immunostaining and weak TUNEL-positive staining in chondrocytes from ACLT-injured knees. Treatment with miR-128a-AS **e** attenuates ACLT-mediated increase in OARSI scores, Atg12, and LC3 immunostaining, as well as chondrocyte apoptosis. **f** Antisense treatment further improved collagen II and aggrecan expressions. Data are means ± SEM calculated from nine rats. Asterisks (*) indicate *P* < 0.05 between groups

As forced miR-128a expression correlated with cartilage destruction, we next investigated the effects of miR-128a antisense oligonucleotide (miR-128a-AS) administration on the progression of knee OA. At 1 week postoperatively, ACLT-affected joints were received intra-articular injections with lentiviruses expressing miR-128a-AS (Fig. 4a). This treatment significantly repressed the ACLT-induced elevation in miR-128a expression in articular chondrocytes in proximity to cartilage lesions (Fig. 4b). At 8 weeks after ACLT, articular cartilage integrity was compromised as evident from increased serum levels of CTX-II and COMP, but significantly improved in the miR-128a-AS-treated group (Fig. 4c). Thus, miR-128a-AS treatment slowed down ACLT-mediated cartilage destruction and significantly decreased OARSI score (Fig. 4d, e). The antisense oligonucleotide treatment also reduced the ACLT-mediated depletion of Atg12 and LC3. Chondrocytes apoptosis was further evident from TUNEL staining (Fig. 4d, e). In addition, knockdown of endogenously expressed miR-128a rescued the ACLT-induced loss in collagen II and aggrecan expression (Fig. 4f). In contrast, mock treatment did not influence ACLT-induced chondrocyte apoptosis, or cartilage erosion.

### miR-128a-AS reduced synovitis, osteophyte formation, and subchondral bone changes

**Fig. 5:**
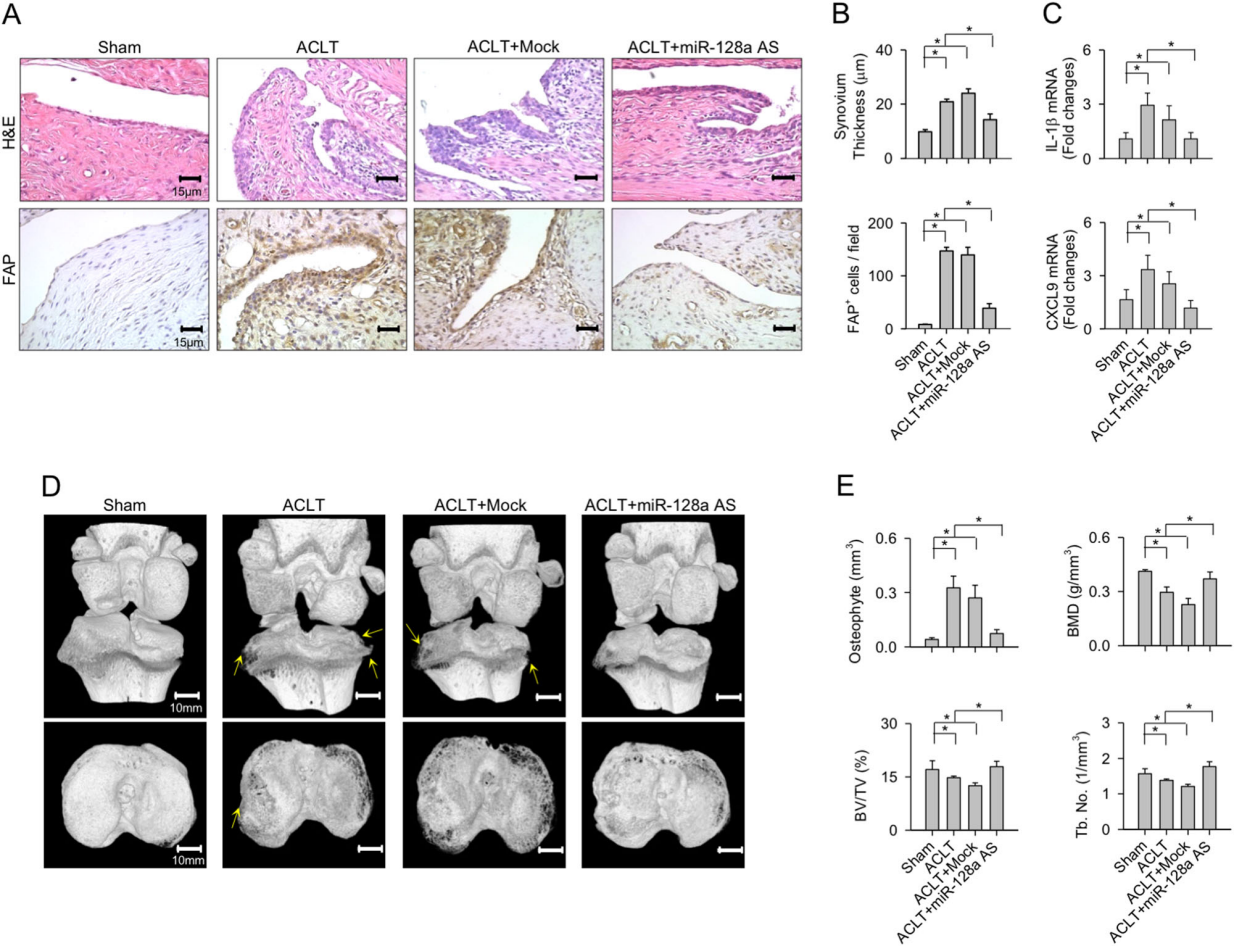
Effects of anti-miR-128a (miR-128aAS) intervention on ACLT-injured osteoarticular tissues. **a** Upon miR-128-AS treatment, synovial tissues show mild synovial lining thickening, and synovial fibroblasts express weak FAP immunoreactivity. Treatment with miR-128a-AS reduced ACLT-mediated disease progression with respect to **b** synovial membrane swelling, fibroblast activation, and **c** IL-1β, and CXCL9 expression. **d** μCT images of osteophytes (yellow arrows) and subchondral plate morphology. **e** Administration with miR-128a-AS repressed ACLT-mediated osteophyte formation, and restored bone mineral density, BV/TV, and Tb.N of the subchondral bone. Data are means ± SEM calculated from nine rats. Asterisks (*) indicate *P* < 0.05 between groups

During the course of OA, synovitis, osteophyte formation, and subchondral bone change occur^25^. We thus evaluated the effects of miR-128a knockdown on these tissues, too. ACLT caused membrane hyperplasia and hypercellularity in the synovial compartment, with a large number of activated synovial fibroblasts staining positive for fibroblast activation protein (FAP). Upon treatment with miR-128a-AS, however, only mild membrane thickening and moderate cell infiltration was visible in the synovium (Fig. 5a). Thus, miR-128-AS treatment significantly reduced ACLT-induced synovium swelling and fibroblast activation (Fig. 5b) as well as and IL-1β, and CXCL9 expression (Fig. 5c).

Furthermore, enhanced osteophyte formation and subchondral bone erosion at the lateral tibial plateau are evident on μCT images of the ACLT-affected articular compartment as compared to the sham group (Fig. 5d). Morphometric analyses further confirmed a significant increase in osteophyte volume together with a decrease in bone mineral density (BMD), trabecular bone volume (BVTV), and trabecular number (Tb.N) in the subchondral bone (Fig. 5e). In contrast, in the miR-128a-treated group, evidence of radiopaque osteophytes was lacking despite mild subchondral plate irregularities (Fig. 5d) and minor subchondral bone loss (Fig. 5e). Mock treatment did not significantly alter ACLT-induced synovium swelling, osteophyte formation, and subchondral bone loss.

### miR-128a reduced survival and cartilage formation capacity of chondrocytic cells

**Fig. 6:**
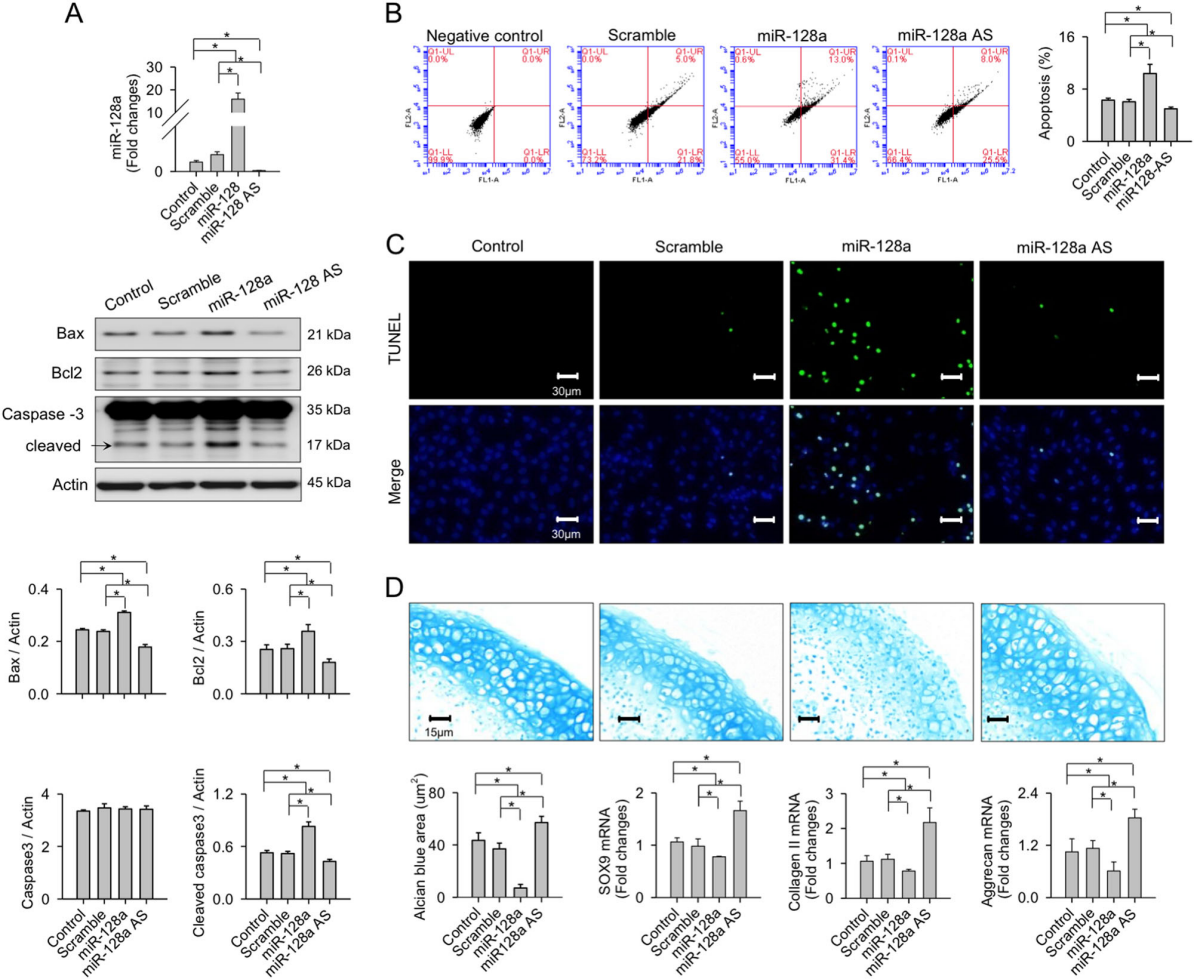
Effects of miR-128a signaling on apoptosis and cartilage formation in vitro. Forced miR-128a expression **a** increased miR-128a expression and levels of Bax, Bcl2, cleaved caspase-3, and **b** apoptosis in chondrocytic cells. **c** A large number of miR-128a precursor-treated chondrocytes displaying strong TUNEL staining. **d** Reduced cartilage ECM synthesis and expressions of SOX9, collagen II, and aggrecan upon miR-128a expression in chondrocyte micro-mass cultures. Note the reduced apoptosis and TUNEL staining upon knockdown of miR-128a, with enhanced cartilage formation by chondrocytes. Data are expressed as the means ± SEM calculated from six repeated experiments. Asterisks (*) indicate *P* < 0.05 between groups

miR-128 is shown to regulate Bax and caspase signaling to accelerate apoptosis^26^. We verified whether miR-128a changed survival or expression of chondrocyte markers in neonatal rat knee cartilage. Transfecting miR-128a precursors significantly increased expression of miR-128a but also increased the amount of apoptosis regulators Bax, Bcl2, and cleaved caspase-3, respectively (Fig. 6a). Furthermore, a large number of miR-128a-transfected cells progressed into apoptosis as evident from annexin-V (Fig. 6b) and TUNEL (Fig. 6c) staining, respectively. Overexpression of miR-128a further significantly reduced cartilage ECM accumulation in micro-mass cultures. A weaker Alcian blue staining and a reduced expression of chondrocytic markers SOX9, collagen II, and aggrecan (Fig. 6d), respectively, is indicative of a significantly reduced ECM synthesis in these cultures upon miR-128a overexpression. In contrast, miR-128a knockdown significantly reduced baseline abundances of Bax, Bcl2, and cleaved caspase-3 (Fig. 6a) as well as apoptosis in vitro (Fig. 6b, c). The miR-128a-AS-treated cell cultures showed higher cartilage forming capacity and chondrocyte-specific marker expression than the controls (Fig. 6d). Apoptosis and chondrocyte marker expression were not altered in the scrambled controls.

### miR-128a targeted the 3′-UTR of Atg12

**Fig. 7:**
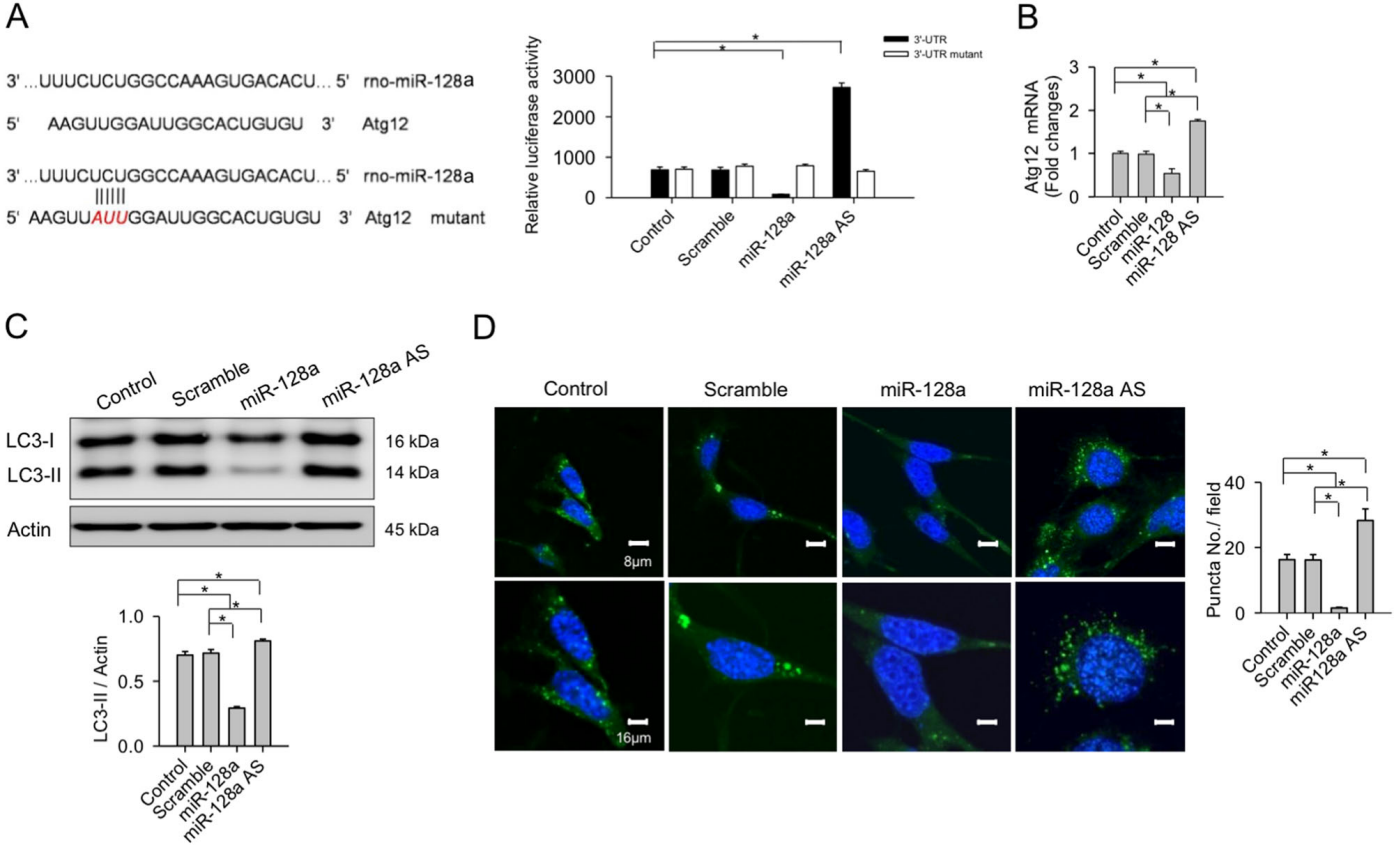
Effects of miR-128a signaling on autophagy in chondrocytes. Overexpression of miR-128a increases **a** luciferase activity of Atg12-3′-UTR luciferase reporter and downregulates **b** Atg12 expression and **c** LC3-II levels as well as **d** autophagic puncta formation. Knocking down miR-128a reduces Atg12-3′-UTR luciferase reporter activity but increases chondrocyte autophagy. Data are means ± SEM. Asterisks (*) indicate *P* < 0.05 between groups

As bioinformatics (www.mirbase.org) predicts that miR-128 targets Atg12 expression, we postulated that miR-128a may directly affect Atg12 mRNA expression in chondrocytes, too. Increasing miR-128 expression significantly reduced the 3′-UTR luciferase reporter activity of Atg12, whereas its knock down reversed this effect (Fig. 7a). In contrast, luciferase activities of the mutated 3′-UTR of Atg12 was not significantly affected by this treatment, suggesting that miR-128a binds directly to the 3′-UTR of Atg12. As a result, increased miR-128a expression significantly reduced Atg12 expression and LC3-II concentrations (Fig. 7b, c). Moreover, abundant autophagic puncta formation was evident from fluorescent monodansylcadaverin probing for autophagic vacuoles (Fig. 7d). In contrast, silencing miR-128a significantly increased Atg12 expression and LC3-II levels as well as abundance of autophagic vesicles (Fig. 7b–d).

### EZH2 methylation of histone H3K27 regulated miR-128a expression

**Fig. 8:**
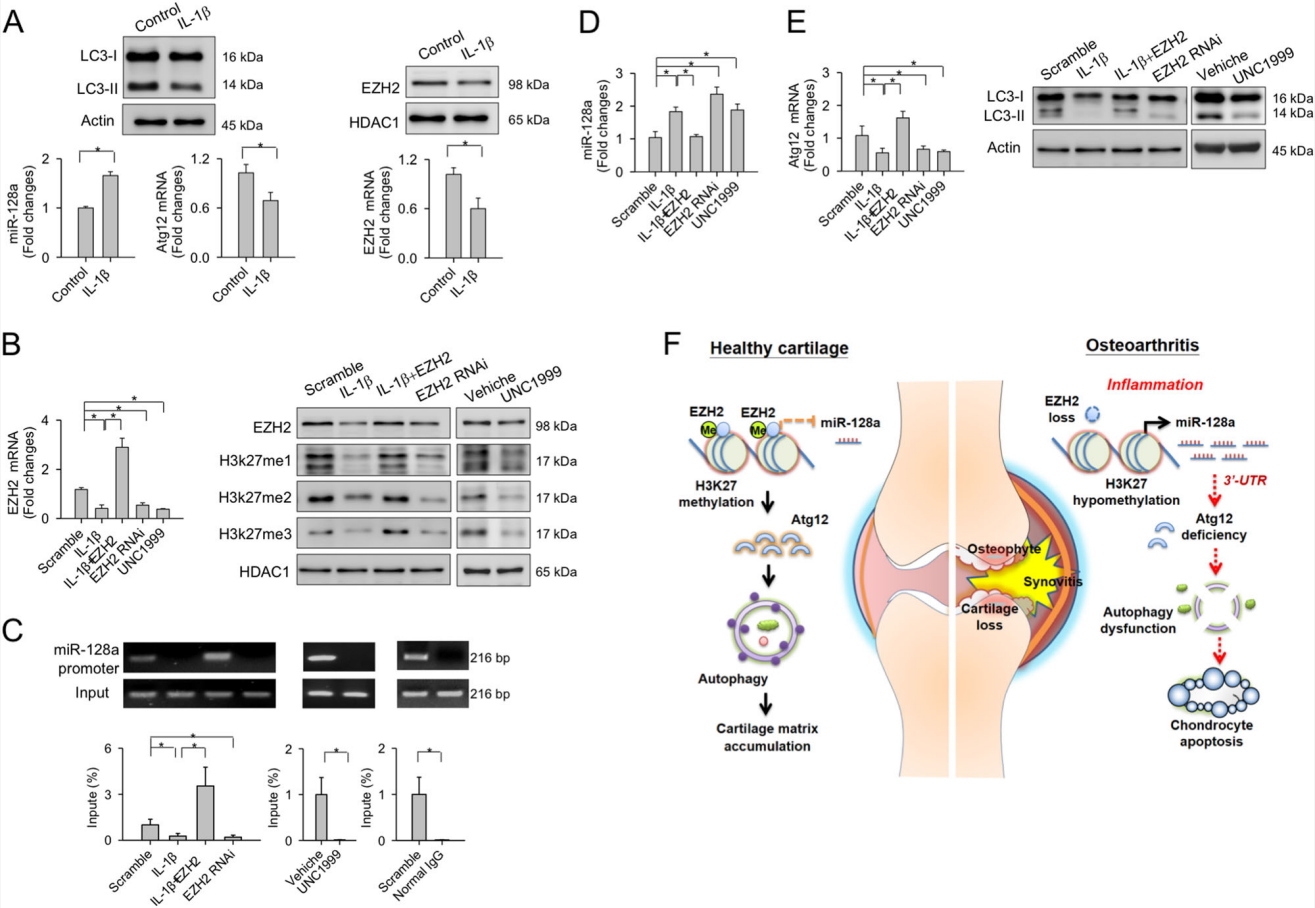
Epigenetic control of miR-128 transcription in inflamed chondrocytes. **a** IL-1β upregulates expression of miR-128a, but represses that of EZH2, Atg12, and LC3-II. Overexpression of EZH2 increases **b** H3K27me1, H3K27me2, and H3K27me3 abundances as well as **c** occupancy of H3K27me2 at the miR-128 proximal promoter regions. Consequently, **d** miR-128a expression is reduced. **e** IL-1β-induced suppression of Atg12, and LC3-II expression with a subsequent loss of LC3-II synthesis. Loss of EZH2 function by EZH2 RNAi or UNC1999 treatment, reverses the H3K27me2 enrichment in the miR-128a promoter region to increase miR-128a expression. In turn, the latter reducing that of Atg12 and LC3-II. Data are means ± SEM calculated from four repeated experiments. Asterisks (*) indicate *P* < 0.05 between groups. **f** Schematic illustration of epigenetic deregulation of miR-128a targeting Atg12 to impair chondrocyte autophagy and to accelerate development of osteoarthritis

Epigenetic deregulation of miR-128a correlates with the incidence of hip OA^22^. We thus tested whether upregulated miR-128a expression can be correlated to histone methylation in inflamed articular chondrocytes. IL-1β treatment of cells in vitro, to mimic inflammation, significantly increased miR-128a expression but reduced Atg12 expression, and LC3-II levels (Fig. 8a). IL-1β-mediated stress also reduced the abundances of histone methyltransferase enhancer zeste homology 2 (EZH2) (Fig. 8a) and mono-methylated, bi-methylated, and tri-methylated H3K27 (Fig. 8b). Forced expression of EZH2 further increased the abundances of methylated H3K27 (Fig. 8b) and H3K27me2 enrichment in the −688 to −427 bp proximal region of the miR-128a promoter (Fig. 8c). This reduced miR-128a expression (Fig. 8d) and attenuated IL-1β-mediated loss of Atg12 and LC3-II (Fig. 8e). ChIP-PCR reactions of IgG immunoprecipitates were undetectable, confirming the specificity of the used H3K27me2 antibody. EZH2 knockdown or pharmacological inhibition using UNC1999 significantly decreased the concentration of methylated H3K27 (Fig. 8b). It also reduced H3K27me2 enrichment to the miR-128a promoter region (Fig. 8c) and elevated miR-128a expression (Fig. 8d), but repressed Atg12 and LC3-II levels (Fig. 8e).

## Discussion

During the development of OA, a plethora of biochemical and mechanical stresses cumulatively disintegrate arrays of intracellular signaling pathways that compromise cartilage, synovium, and subchondral bone integrity and favor disease progression^25^. It is highly demanding to develop molecular modalities for attenuating the impact of this degenerative joint disorder^27^. MicroRNAs are known to modulate progenitor cell survival^28^, and enhance metabolic programming and tissue repair^29^. Still little is known about how microRNA signaling may affect chondrocyte behavior during the pathogenesis of OA. To our best knowledge, this study is the first to uncover a role of miR-128 signaling in the development of gonarthrosis. We show that miR-128a hinders chondrocyte autophagy, alters the joint microenvironment and thus its homeostasis to aggravate cartilage erosion. Our analyses further highlight the efficacy of miR-128a knockdown strategies to modulate this cartilage destruction. Moreover, anti-miR-128a appears to protect synovium and the subchondral bone integrity to ultimately drag the progression of OA.

Deregulation of chondrocyte autophagy and microRNA signaling are reported to occur in injured articular cartilage during the course of OA. Our results are in agreement with previous studies demonstrating that impaired autophagy in chondrocytes accelerates cartilage destruction^30,31^. In addition, we revealed that ACLT-mediated stresses result in the differential expression of 14 microRNAs in articular chondrocytes, each of which may exert distinct effects on chondrocyte fate and reflect the intricate nature of deteriorative processes during the development of OA. Of these differently expressed microRNAs, miR-128a abundance was most prominently increased in the injured cartilage and coincided with aberrant autophagy in chondrocytes.

Our present study uncovered that high levels of miR-128a expression correlate with end-stage human knee OA. This agrees with earlier reports, where DNA hypomethylation mediated miR-128 overexpression and correlates with Mankin scores of cartilage erosion in osteoarthritic hips^22^. This observation is consistent with data on experimentally induced, ACLT-mediated knee OA, development in rats. Forced miR-128a expression in the articular compartment further resulted in histopathologic features of OA, like cartilage matrix degradation and severely altered articular morphology. Our current investigations strongly indicate that miR-128a is detrimental to chondrocyte homeostasis and cartilage integrity. We also showed that miR-128a negatively affected cartilage development from chondrocytic cells in micro-mass culture in vitro. This is a further indication of its adverse biological function to cartilage anabolism.

Age is one of important cause of OA prevalence^2^. Supra-physiological apoptosis in aging chondrocytes^32^, and in destabilized meniscus surgery-induced cartilage damage^33^ are linked to the development of knee OA. miR-128 signaling has been found to regulate autophagy, apoptosis, and differentiation of various cell types in physiological and pathological statuses^34–36^. We showed that miR-128a disturbed autophagy and drove chondrocytes towards excessive apoptosis. To this end, data from our cell culture experiments were consistent with those from our ACLT-mediated cartilage damage in vivo. Our data are also in agreement with studies by other groups demonstrating that impaired autophagy enhances apoptosis in osteoarthritic chondrocytes affected by glucocorticoids^13^ or oxidative stresses^37^, respectively. Our study now suggests a novel microRNA mechanism underlying the defective autophagy that enhances chondrocyte apoptosis during the progression of OA. We have shown that miR-128a targeted the 3′-UTR of Atg12 in these cells. This latter autophagy regulator is indispensable for the elongation of the autophagosome membrane^38^. Consequently, we proposed that miR-128a blocks autophagy in these cells, which is in line with reports that proteasomal degradation of Atg12 causes apoptosis in various other cell types^39^. Decreasing the Atg12-Atg5 conjugate is further known to hinder cartilage development^40^. Our current data thus offers novel molecular insights into how miR-128a deregulates Atg12 stabilization and disintegrates autophagic and apoptotic processes in chondrocytes during the course of OA development.

The detrimental role of miR-128a signaling during the development of OA led us to further explore the intracellular pathways contributing to its elevated expression in more detail. To this end, IL-1β-stressed chondrocyte cultures served as a simplified model to simulate OA-like effects in vitro^41^. Epigenetic H3K27 methylation is another contributing factor in cartilage development in craniofacial tissues^42^ and a regulator of metabolic activity of osteoarthritic chondrocytes^43^. Methylation of lysine 27 of histone 3 (H3K27) results in transcriptional repression. Enhancer of zeste homolog 2 (EZH2), a subunit of the polycomb repressive complex 2 (PRC2) participates in regulating this repressive H3K27me mark^44^. Intriguingly, our data now suggest that IL-1β elevates miR-128 transcription through repressing EZH2-mediated regulation of H3K27 methylation and removing the H3K27me2 repressive mark in the miR-128 promoter region and thereby increasing its transcriptional accessibility. This is in line with earlier reports showing that loss of EZH2 function in chondrocytes impairs hypertrophy and growth plate chondrogenesis^45^. EZH2 depletion in inflamed chondrocytes would also explain the dysfunctional chondrocytes in osteoarthritic joints. Our data are a first indication that an epigenetic interplay between microRNA and histone methylation modulates intracellular activities and cell fate of articular chondrocytes during OA progression. Epigenetic regulation of OA development seems worth further characterization in the future. At present, we cannot exclude the possibility that other microRNAs or histone methylation regulators may also contribute to autophagy or survival of osteoarthritic chondrocytes.

Controlling microRNA signaling is a promising new regime to reduce pathologically increased remodeling activities, and subsequently promote regenerative processes in various joint disorders^46–48^. Manipulating miR-128a signaling to attenuate joint disorders, however, has not yet been previously explored. The apparent importance of miR-128a for autophagic activities and cartilage matrix metabolism in chondroctyic cells prompted us to study the remedial effect of anti-miR-128a therapies on disease development. Upon ACLT-induced OA progression, miR-128a-AS (i.e., antisense) treatment improved the condition of the most relevant knee joint tissues, altering autophagic puncta loss, chondrocyte apoptosis, and articular gross morphology. MicroRNA-128a antisense treatment further reduced serum levels of CTX-II and COMP, which is indicative of reduced cartilage erosion. With synovitis^49^ and subchondral bone alterations^50^ simultaneously occurring in OA joints, it is noteworthy that miR-128a-AS therapy also improved synovial membrane hyperplasia and fibroblast activation. Additionally, osteophyte formation and subchondral plate destruction were also beneficially influenced by the miR-128a-AS treatment. Compromised integrity of subchondral bone microarchitecture is known to contribute to cartilage loss^51^ and synovitis^52^ during OA development. The beneficial responses of the synovium and subchondral bone to the miR-128a-AS treatment in ACLT-injured joints is further in line with an overall preservation of joint homeostasis by miR-128a suppressive therapies.

Our results of shed a new light on the deleterious role of miR-128a expression during the OA pathogenesis and indicates an Atg12-dependent deregulation of chondrocyte autophagy as an underlying cause. As EZH2 regulation of H3K27 methylation controls miR-128a transcription in inflamed chondrocytes (Fig. 8f). our findings pave the road for potential therapeutic applications of miR-128a suppressive strategies to prevent the progression of knee OA.

## Materials and methods

### ACLT-mediated knee OA

Experimental protocols for laboratory animals were approved by the IACUC of Kaohsiung Chang Gung Memorial Hospital (Nos. 2012020801 and 2015061701) and conducted in a specific pathogen-free vivarium. One hundred and eight male Sprgaue-Dwaley rats (16 weeks old) were evenly divided into sham and ACLT groups. Of the 27 rats in each group, 9 animals were randomly selected for histomorphometry, 9 rats were employed for immunoblotting, and 9 rats were selected for PCR assay. After anesthesia, the animals’ left knees were subjected to medial parapatellar arthrotomy and ACLT or sham surgery using aseptic surgical procedures, as previously described^53^. At 4 weeks and 8 weeks postoperatively, the animals were killed, and the affected joints were dissected for study.

### Clinical specimens

For collection of clinical specimens, ethical approval was obtained from the Institutional Review Board of Chang Gung Memorial Hospital (Approval No 104-5248B). After written informed consent was obtained, 28 patients with end-stage OA of the knee (23 females and 5 males; 71 ± 1.1 years) requiring total knee arthroplasty, were included as OA group. Seventeen patients (13 females and 4 males; 54.1 ± 5.8 years) with femoral neck fracture receiving hip arthroplasty were enrolled as non-OA group. Articular cartilage was harvested from the injured joints after osteotomy for arthroplasty.

### microRNA profiling

Articular tissues from injured rat joints were dissected using a surgical microscope. Specimens were homogenized using a Precellys 24^®^ homogenizer and a liquid nitrogen cooling system (Bertin Technologies, Montigny-le-Bretonneuz, France). Total microRNA was then extracted from the homogenates using the Biochain^®^ MicroRNA Isolation Kits (Biochain Institute Inc, Newark, CA). MicroRNA expression was detected using Megaplex^TM^ Pool arrays (Applied Biosystems Inc, Foster City, CA), according to the manufacturer’s instructions. Next, 1 μg of total microRNA was reverse transcribed by MultiScribe^®^ reverse transcriptase, 10×Megaplex^TM^ RT primers, dNTP and 10×RT Buffer and templates and further incubated with 2×TaqMan^®^ PreAmp Master Mix and 10×Megaplex^TM^ PreAmp Primers using an ABI 7900 Detection System (Applied Biosystems Inc, Foster City, CA). The start of the logarithmic amplification of the PCR reactions was computed automatically and interpreted as cycle threshold (Ct). Relative expression was calculated according to Eq. 2^−ΔΔCt^, where ΔΔCt = ΔCt_ACLT_ −ΔCt_sham_ and ΔCt = Ct_microRNA_ − Ct_U6,_ was adapted to quantify changes in microRNA expression in the ACLT group. Potential microRNA candidates were selected based on an at least fivefold change cutoff in relative expression.

### Lentiviral miR-128 precursors and antisense oligonucleotide

Expression vectors pMIF-cGFP-zeo (System Biosciences, Palo Alto, CA) encoding miR-128a, and pmiR-ZIP-shRNA encoding miR-128a antisense oligonucleotides (miR-128a-AS) (AM11746; Applied Biosystems Inc, Foster City, CA) were constructed, respectively. Expression vectors and pPACKF1 vectors were transferred into 293T cells. Lentiviral particle titers (infectious units/ml) were quantified using LentiX qRT-PCR Titration Kits (Clontech Laboratories Inc, San Francisco, CA) following enrichment at 1,000,000×*g* for 60 min at 4 °C. Ten μl of aliquots of lentiviral particle mixtures containing 1 × 10^9^ infectious units/ml were then prepared for intra-articular injection.

### Intra-articular injections with lentiviral miR-128a or miR-128a-AS

Rats received miR-128a (*n* = 9) or mock control (*n* = 9) via intra-articular injection, while control animals (*n* = 9) received vehicle only. In some experiments, ACLT-treated rats were treated with miR-128a-AS (*n* = 9), mock (*n* = 9), or vehicle (*n* = 9) at 1 week postoperatively, while 9 rats receiving sham surgery (normal control group). Animals were killed and knee joint dissected at 8 weeks postoperatively.

### Quantification of cartilage breakdown products in sera

Peripheral blood serum fractions were isolated; and levels of C-terminal crosslinking telopeptide of type II collagen (CTX-II), and cartilage oligomeric matrix protein (COMP) were measured using respective ELISA kits (Nordic Bioscience A/S, Herlev, Denmark), normalized to the protein concentrations of the sera.

### MicroCT analysis

Knee joints were scanned using a Skyscan 1176 µCT scanner (Bruker, Kontich, Belgium) with the following setting: 50 keV X-ray energy, 500 μA intensity, and a 69 ms exposure time. Each image with an isotropic 9 μm voxel size was obtained. Overall, 400 transverse images of the region of interest between the articular end and growth plate subjected to reconstruction into three dimensional images using SKYSCAN^®^ CT-Analyser software. Osteophyte formation and subchondral plate microstructure were evaluated by an orthopedic surgeon blind to the treatment. The osteophyte volume (mm^3^) and trabecular bone mineral density (BMD, g/mm^3^), volume (BV/TV, %), and number (Tb.No, 1/mm) of subchondral bone regions in the proximal tibiae were calculated automatically.

### Histomorphometry, in situ hybridization, and immunohistochemistry

Safranin-O staining of sections was performed according to histochemical protocol (Sigma-Aldrich). The severity of articular cartilage injury at the proximal tibiae of 10 sections spanning 400 µm was scored using the OASRI and Mankin scales. Histomorphometry of articular tissue and thickness of the synovial membrane were analyzed using a Zeiss microscope and image analysis system. Twenty-four sections from 8 rats were randomly selected for quantification. miR-128a transcripts in sections were detected by Biochain^®^ IsHyb In Situ Hybridization kits (Biochain Institute Inc, Newark, CA) and digoxigenin-labeled miR-128a probes (Applied Biosystems Inc, Foster City, CA), according to the manufacturer’s instructions. Where applicable, immunostaining was performed using an immunohistochemistry detection kits (BioGenex, Fremont, CA) and monoclonal antibodies for fibroblast activation protein (FAP), LC3, and Atg12 (Cell Signaling, Danvers, AM). Apoptotic cells in sections were probed using In Situ TUNEL Detection Kits (Roche Diagnostics GmbH). Numbers of chondrocytes and synovial fibroblasts positive for FAP, LC3, and Atg12 immunoreactivity, and TUNEL staining were counted in each field; and 32 fields in 16 sections from 8 rats were analyzed.

### Chondrocyte cultures

Seven-day-old rats were subjected to euthanasia and articular cartilage of the knees were dissected, as previously described^54^. Chondrocytes were then isolated using routine collagenase digestion and incubated in DMEM and 10% fetal bovine serum. Cell cultures (5 × 10^5^ cells per well, 6-well plates) were transfected with a mixture of transfection agent Lipofectamine^TM^ 2000 (Invitrogen), 50 nM miR-128a precursor, antisense oligonucleotide, and scramble control (Applied Biosystems Inc, Foster City, CA), according to the manufacturer’s instructions. For micro-mass cultures, aliquots of 2 × 10^6^ cells were centrifuged to form cell pellets, followed by incubation at 37 °C for 7 days. In some experiments, cell cultures were transfected with vectors encoding EZH2 or RNAi (S65775; Applied Biosystems Inc, Foster City, CA) or treated with 4 μM UNC1999 (Sigma-Aldrich), an EZH2 inhibitor, for 24 h and further incubated in medium containing 10 ng/ml IL-1β (R&D Systems, Minneapolis, MN) for 24 h.

### Assessment of cell apoptosis

Chondrocytes were treated with miR-128a precursor, antisense oligonucleotide, and scrambled control and the number of apoptotic cells per 10^5^ cells in total was quantified using flow cytometry (BD Accuri C6, San Jose, CA) upon probing with Annexin-V-FITC Apoptosis Detection Kits (Enzo Life Sciences Inc., Farmingdale, NY). In a subset of experiments, apoptotic cells were confirmed using fluorescent TUNEL staining (Roche Diagnostics GmbH), according to the manufacturer’s instructions.

### Luciferase reporter activity

The wild-type sequence (5′-AAGUUGGAUUGGCACUGUGU-3′; NM_001038495.1) and three-base mutation sequence (5′-AAGUUAUUUUGGCACUGUGU-3′) of the 3′-UTR within Atg12 putatively binding to miR-128a were ligated into pCRII-TOPO II luciferase receptor vectors (Invitrogen), respectively. Chondrocytes (1 × 10^4^ cells per well, 96-well plate) were co-transfected with 10 ng luciferase reporter vectors and 10 ng nM Renilla luciferase reporter vector and followed by transfecting with 30 nM miR-128a precursor, antisense oligonucleotide, or scramble control. Luciferase activities in cell cultures were quantified using a Dual Luciferase Detection Kits (Promega) and normalized with Renilla luciferase activity.

### RT-quantitative PCR

Total RNA from articular cartilage and chondrocyte cultures was isolated using QIAzol reagent (Qiagene). One μg total RNA was subjected to reversed transcription. Amplification was performed using a ABI 7900 Detection System (Applied Biosystems), with 2 × TaqMan^®^ Universal PCR Master Mix. Primers for Atg4, Atg12, p62, Beclin, collagen II, aggrecan, SOX9, IL-1β, CXCL9, and calibrator gene 18S rRNA (Supplementary Table 1). Changes in mRNA expressions were calculated as 2^−ΔΔCt^, where ΔΔCt = ΔCt_ACLT_ – ΔCt_sham_ and ΔCt = Ct_gene_ − Ct_18S_.

### Immunoblotting

Protein extracts of articular tissues or chondrocyte cultures were isolated using Mammalian Cell Extraction Kit (Abcam, Cambridge, MA). After electrophoresis and blotting, proteins of interest were probed with LC3, Bax, Bcl2, caspase-3, EZH2, H3K27me1, H3K27me2, H3K27me3, and actin antibodies (Cell Signaling Technology, Danvers, MA). Secondary antibody IgG conjugated horseradish peroxidase (Cell Signaling Technology) incubation, and LumiGLO^®^ chemiluminescent agent in presence of hydrogen peroxide were used for detection, according to the manufacturer’s instructions.

### Chromatin immunoprecipitation (ChIP)-PCR

H3K27me2 in vehicle-, IL-1β-, EZH2 cDNA-, EZH2 RNAi-, and UNC1999-treated chondrocyte cultures (5 × 10^6^ cells) was immunoprecipitated using H3K27me2 antibody, IgG, and Megna ChIP™ A/G Chromatin Immunoprecipitation (ChIP) kits (Millipore, Temecula, CA), according to the manufacturer’s instructions. To isolate DNA, the immunoprecipitates were subjected to sonication, elution, Proteinase K digestion, and enrichment with column elution. For PCR assessment, aliquots of DNA specimens were pipetted with Cy3-conjuated probes (forward: 5′-ACGACAGATTGAAGGCCTGGG-3′; reverse: 5′-GGTGCTCTTCCCCAATCAT-3′) (Applied Biosystems) for the −688 to −427 bp region proximal to the transcription start site of the miR-128a promoter (Ensembl:ENSG00000207654). The positive control GADPH promoter was detected using another Cy3-conjugated probe (forward: 5′-TACTAGCGGTTTTAC GGGCG-3′; reverse: 5′-TCGAACAGGAGGAGCAGAGAGCGA-3′). To test the amplification efficiency, standard curves of Ct values vs. serial fivefold dilutions of DNA were plotted and H3K27me2 enrichment to the miR-128a promoter was expressed as % input DNA.

### Statistical analysis

All data were displayed as means ± SEM. Analyses of the microRNA array in the ACLT and sham groups, as well as investigations in the OA and non-OA groups, were verified by the Wilcoxon test. Normal control, ACLT, miR-128a, and miR-128a-AS groups were analyzed by a parametric analysis of variance and a Bonferroni post hoc test. Statistical differences were indicated by *P-*values lower than 0.05.

## Electronic supplementary material

Supplementary Table 1

Fig. S1

Supplementary figure legends
