# Supplementary material for: MicroRNA-128a represses chondrocyte autophagy and exacerbates knee osteoarthritis by disrupting Atg12
Source: Cell Death Dis. 2018 Sep 11;9(9):919. doi: 10.1038/s41419-018-0994-y (PMC6134128; doi:10.1038/s41419-018-0994-y)
Supplement: Supplementary file 1 — Supplementary Table 1 [file 41419_2018_994_MOESM1_ESM.docx]

**Supplementary Table 1**

**Oligonucleotide sequences**

| **Oligonucleotide sequences and RT-qPCR primers for human specimens** | |
| --- | --- |
| miR-128a | UCACAGAACCGGUCUCUUU |
| U6 | GTGCTCGCTTCGGCAGCACATATACTAAAATTGGAACGATACAGAGAAGATTAGCATGGCCCCTGCGCAAGGATGACACGCAAATTCGTGAAGCGTTCCATATTTT |
| Atg12 | Forward: 5’-CGGGAACACCAAGTTTCACT-3’  Reverse: 5’-TTGTTTCTGGGGAGACATCC-3’ |
| β-Actin | Forward: 5’-GTAACCCGTTGAACCCCATT-3’  Reverse: 5’-CCATCCAATCGGTAGTAGCG-3’ |
| **Oligonucleotide sequences and RT-qPCR primers for rat specimens** | |
| miR-128a | UCACAGAACCGGUCUCUUU |
| U6 | GTGCTCGCTTCGGCAGCACATATACTAAAATTGGAACGATACAGAGAAGATTAGCATGGCCCCTGCGCAAGGATGACACGCAAATTCGTGAAGCGTTCCATATTTT |
| Atg4 | Forward: 5’- ACACAGGGTTGATTTGGTTTATG-3’  Reverse: 5’- ATTGCTTCCAGTCAGAGTAAGG-3’ |
| Atg12 | Forward: 5’- CACAGCATTTGCCGTACTGT-3’  Reverse: 5’- CTCTGTCACCATTGCTCTTC-3’ |
| p62 | Forward: 5’- GGTGGAGGGTGCTTTGAATA-3’  Reverse: 5’- GATGCTGTCCTGGGTTTCT-3’ |
| Beclin | Forward: 5’- TGCCTCCAGTGTCTTCAATC-3’  Reverse: 5’- GTGTGCAGCAGTTCAAAGAAG-3’ |
| Collagen II | Forward: 5’-GCAAGGAGAAGAAGCACATC-3’  Reverse: 5’-GGACAGTAGACGGAGGAAAG-3’ |
| Aggrecan | Forward, 5’-ATCAAGTGGAGCCGTGTTTC -3’;  Reverse: 5’-CAGAGTCATTGGAGCGAAGG-3’ |
| SOX9 | Forward: 5’-GCCACCTGCAACTGTGATTA-3’  Reverse:5’-CACAGCATTTGCCGTACTGT-3’ |
| EZH2 | Forward: 5’-ATGTGAGAAGGGACCGGTTT-3’  Reverse: 5’-CTGTATCCTTCGCTGCTTCC -3’ |
| IL-1β | Forward: 5’-AGCTTTCAGCTCACATGGGT-3’  Reverse: 5’-CAGGAAGGCAGTGTCACTCA-3’ |
| CXCL9 | Forward: 5’-AGCAAGGGTGAAGCTGA-3’  Reverse: 5’-GCGCTTGTTGGTAAAGTGGT-3’ |
| 18S rRNA | Forward: 5’-GTAACCCGTTGAACCCCATT-3’  Reverse: 3’-CCATCCAATCGGTAGTAGCG-5’ |
