# Supplementary figures and images for: MicroRNA-128a represses chondrocyte autophagy and exacerbates knee osteoarthritis by disrupting Atg12
Source: Cell Death Dis. 2018 Sep 11;9(9):919. doi: 10.1038/s41419-018-0994-y (PMC6134128; doi:10.1038/s41419-018-0994-y)

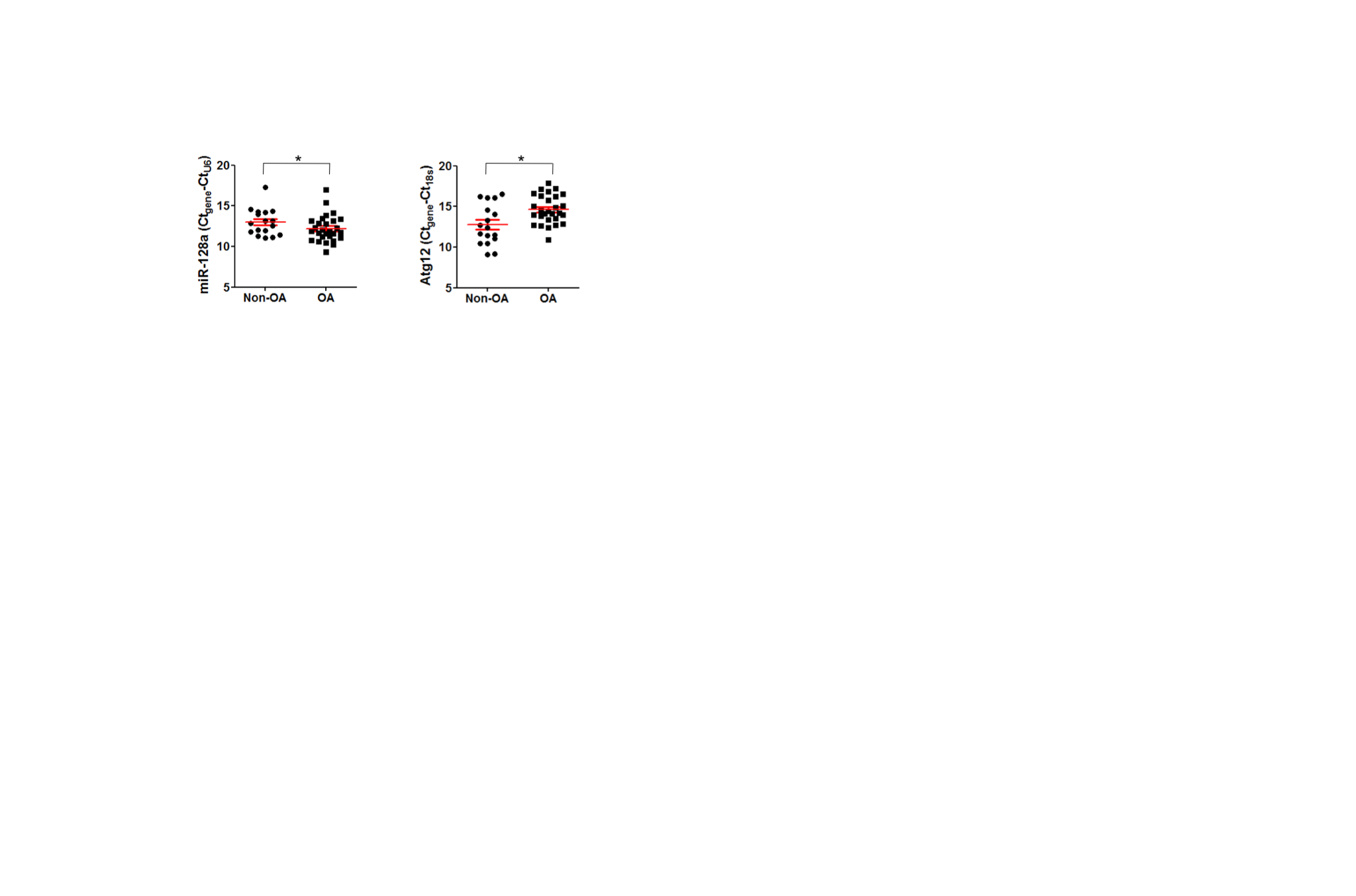

Supplement: Supplementary file 2 — Fig. S1 [file 41419_2018_994_MOESM2_ESM.tif]
