# Supplementary material for: MicroRNA-128a represses chondrocyte autophagy and exacerbates knee osteoarthritis by disrupting Atg12
Source: Cell Death Dis. 2018 Sep 11;9(9):919. doi: 10.1038/s41419-018-0994-y (PMC6134128; doi:10.1038/s41419-018-0994-y)
Supplement: Supplementary file 3 — Supplementary figure legends [file 41419_2018_994_MOESM3_ESM.docx]

**Supplementary figure legend**

Fig. S1 RT-qPCR analyses for miR-128a and Atg12 expression of articular cartilage from OA and non-OA donors. Expression of miR-128a and Atg12 mRNA was expressed as Ct = Ct_miR128a_ – Ct_U6_ and ΔCt = Ct_Atg12_ – Ct_18S_, respectively. Data are calculated from 28 patients with end-stage knee OA and 17 patients with femoral neck fracture (non-OA). Asterisks (*) indicate *p* < 0.05 between groups.
